# Redox‐Active Separators for Lithium‐Ion Batteries

**DOI:** 10.1002/advs.201700663

**Published:** 2017-12-19

**Authors:** Zhaohui Wang, Ruijun Pan, Changqing Ruan, Kristina Edström, Maria Strømme, Leif Nyholm

**Affiliations:** ^1^ Department of Chemistry‐Ångström The Ångström Laboratory Uppsala University Box 538 SE‐751 21 Uppsala Sweden; ^2^ Nanotechnology and Functional Materials Department of Engineering Sciences The Ångström Laboratory Uppsala University Box 534 SE‐751 21 Uppsala Sweden

**Keywords:** capacity, cellulose, conducting polymers, lithium‐ion batteries, redox‐active separators

## Abstract

A bilayered cellulose‐based separator design is presented that can enhance the electrochemical performance of lithium‐ion batteries (LIBs) via the inclusion of a porous redox‐active layer. The proposed flexible redox‐active separator consists of a mesoporous, insulating nanocellulose fiber layer that provides the necessary insulation between the electrodes and a porous, conductive, and redox‐active polypyrrole‐nanocellulose layer. The latter layer provides mechanical support to the nanocellulose layer and adds extra capacity to the LIBs. The redox‐active separator is mechanically flexible, and no internal short circuits are observed during the operation of the LIBs, even when the redox‐active layer is in direct contact with both electrodes in a symmetric lithium–lithium cell. By replacing a conventional polyethylene separator with a redox‐active separator, the capacity of the proof‐of‐concept LIB battery containing a LiFePO_4_ cathode and a Li metal anode can be increased from 0.16 to 0.276 mA h due to the capacity contribution from the redox‐active separator. As the presented redox‐active separator concept can be used to increase the capacities of electrochemical energy storage systems, this approach may pave the way for new types of functional separators.

## 1 Introduction

Lithium‐ion batteries (LIBs) play a major role in powering portable electronic devices and have also made their way into the electric vehicle (EV) market. However, to meet this market's particular requirements for energy storage, the energy density, and power density of LIBs need to be further improved. This need has resulted in a search for new anode and cathode materials.^1^, ^2^, ^3^ The development of high capacity cathodes has received particular attention, since the capacities of the LIBs generally are limited by the capacities of the cathodes. Contemporary cathode materials (e.g., LiFePO_4_, LiCoO_2_, Li_2_MnO_4_) used in commercial LIBs thus have relatively low theoretical capacities ranging from 140 to 170 mA h g^−1^. While various new cathode materials and electrode‐engineering technologies have been developed to enhance the performance of the batteries,^4^, ^5^, ^6^, ^7^, ^8^ little effort has been made to improve the battery capacities by modifying other parts of the cells other than the electrodes.

The separator, one of the critical LIB components, is a porous insulating membrane placed between the cathode and the anode to prevent their direct electrical contact and ensure sufficient ionic conductivity between the electrodes via the electrolyte present in the pores of the separator.^9^, ^10^ As the function of the separator is crucial to the overall battery performance, including the lifetime, safety, as well as the energy and power densities of the battery, much research has been carried out to identify the best possible separator material. The commercial separators used in state‐of‐the‐art LIBs are typically made of polyolefin and therefore exhibit poor electrolyte wettabilities and low thermal stabilities.^11^ Although surface modifications, based on ceramic and polymer coatings, have been employed to mitigate these problems, the applicability of these modified separators is limited due to manufacturing costs and technical challenges.^12^, ^13^, ^14^, ^15^ This issue have resulted in an increased interest in the development of alternative, inexpensive, and renewable separator materials such as cellulose. Cellulose‐based separators hold great promise due to their inherent hydrophilicity, tailorable structural and mechanical properties, good thermal stability, and straightforward manufacturing procedure.^16^, ^17^, ^18^, ^19^ However, to date, the work on cellulose‐based separators has mainly been focused on the development of safe separators with good electrolyte wettabilities. Hence, very few studies have been published on the possibilities of manufacturing chemically functionalized cellulose separators, which also may increase the capacities of the batteries.^20^ It should also be mentioned that the modification of polyolefin separators and new biopolymer‐based separators generally yields separators with increased thicknesses and decreased porosities (see Table S1 in the Supporting Information for a review of the thicknesses of modified separators reported recently), which may result in deteriorated electrochemical performance and decreased volumetric/gravimetric energy densities of the batteries.

The separators used in commercial LIBs usually have a thickness of ≈25 µm, although separators with a thickness of ≈40 µm are common in EV/Hybrid EV applications^11^, ^21^ to provide proper mechanical strength and sufficient amounts of electrolyte. The volume of the separator, hence, constitutes a significant fraction of the total volume of the LIB, i.e., nearly 20% for cells containing a graphite anode and a LiCoO_2_ cathode.^22^ The volumetric capacities of LIBs could therefore be increased if thinner separators and/or thicker electrodes are used.^3^ While both approaches are plausible, the use of a thinner separator may decrease the mechanical strength and thus increase the risk of separator punctures (which could yield short‐circuits) during the operation of a battery.^21^ A thicker electrode could, on the other hand, adversely impair battery performance due to increased mass transport limitations. Therefore, there is a need for the development of new approaches that facilitate the attainment of increased battery capacities while still maintaining the function of conventional separators.

**Figure 1:**
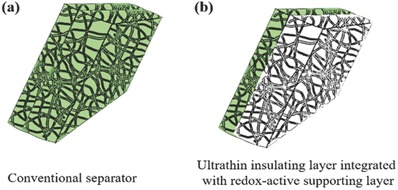
Schematic side‐view illustrations of a) a conventional separator and b) a redox‐active separator. The light green regions represent the insulating material, while the light gray region represents the redox‐active component.

One strategy could be to combine a thin insulating layer with a porous supporting layer composed of conductive and redox‐active materials (seen **Figure** **1**) to obtain a flexible redox‐active separator with a thickness similar to that of a conventional separator. It may then be possible to increase the volumetric capacity of the battery while still ensuring the safe operation of the battery. This approach can be realized by replacing a fraction of the insulating material of a conventional separator with a highly porous redox‐active material that not only offers extra capacity but also helps to maintain the flexibility and mechanical strength of the insulating separator layer. As it is challenging to coat an electrically conductive redox‐active supporting layer on an insulating membrane to manufacture a separator with sufficient mechanical strength, porosity, and thickness, it is not surprising that few such attempts have been made so far.

Herein, we present a flexible mesoporous redox‐active separator composed of nanocellulose fibers (NCFs) and polypyrrole (PPy) composite and fabricated using a straightforward paper‐making process. The redox‐active separator features a bilayered structure where one side comprises a (≈3 µm) thick insulating NCF layer, while the other side is composed of a 7 µm thick redox‐active PPy/cellulose composite layer. While the NCF layer provides the main insulation between electrodes, the redox‐active PPy/cellulose composite layer provides mechanical support to the NCF layer as well as extra capacity to the LIBs. Although thicker redox‐active separators can be straightforwardly manufactured using the same approach, we mainly focus on the design described above to demonstrate the possibility to readily manufacture separators with very thin NCF layers. The results show that the flexible redox‐active separator has significant advantages over commercial polyethylene separators in terms of thermal stability and electrolyte wettability. It is also demonstrated that the redox‐active separator functions as intended, i.e., no short‐circuits were observed during the cycling of proof‐of‐concept cells, and there was a significant capacity increase due to the presence of the PPy‐containing layer. A proof‐of‐concept LIB containing a LiFePO_4_ (LFP) cathode and a redox‐active separator is shown to exhibit a capacity of 67 µAh cm^−3^ (or 81 mA h g^−1^) based on the total volume (or weight) of the separator and cathode. As these values are higher than those (i.e., 18 µAh cm^−3^ or 26 mA h g^−1^) obtained with a conventional separator, the redox‐active separator approach provides a new way of increasing the capacities of conventional LIBs merely via the replacement of the separator.

## 2 Results and Discussion

### 2.1 Fabrication of Redox‐Active Separators

**Figure 2:**
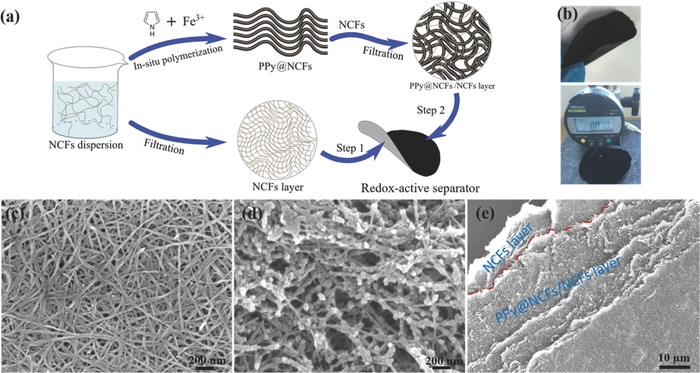
Fabrication and morphological characteristics of the redox‐active separator. a) Schematic illustration of the preparation of the redox‐active separator. b) Photos of the flexible redox‐active separator. c) SEM image of the NCF layer. d) SEM image of the PPy‐containing layer.e) SEM image of a torn redox‐active separator.

The flexible, redox‐active separators were fabricated from nanocellulose using a straightforward paper‐making approach. First, a diluted NCF solution was vacuum‐filtered through a filter membrane to obtain an initial NCF layer on the filter. A second dispersion containing both NCFs and PPy@NCFs, made by the in situ polymerization of pyrrole monomers onto NCFs,^23^, ^24^ was then added and filtered to form a PPy‐containing layer on top of the pure NCF layer (see **Figure** **2** and the Experimental Section). A Fourier transform infrared spectrum for the PPy@NCFs, confirming the presence of PPy is shown in Figure S1 in the Supporting Information. The bilayer membrane was then dried, peeled off the filter membrane and used to fabricate the separators, as is described below. At this stage, the redox‐active separators, which could be made as thin as 10 µm, had a glossy, black, paper‐like appearance and exhibited good mechanical flexibility (Figure 2b). Scanning electron microscopy (SEM) studies indicated the presence of an open mesoporous structure in both the NCFs and the PPy‐containing layer (Figure 2c–e), although some differences could be seen at the micro‐ and nanoscales. The NCFs in the NCF layer had diameters of ≈20–30 nm and were densely packed while the PPy@NCF layer was more porous, containing fibers with diameters of ≈80 nm, in good agreement with our previous results (Figure S2, Supporting Information).^23^ The PPy‐containing layer also appeared to consist of an interconnected fibrous structure in which the NCFs randomly pierced through a web‐like PPy@NCF structure.

In this separator architecture, the ≈3 µm thick, mesoporous NCF layer acts as a separator in the traditional sense by physically separating the cathode and anode in the batteries. Note that an increase in the thickness of the NCF layer results in a decrease in the pore size and porosity of the NCF separator. This results in an increased mass transport resistance and hence decreased battery performance.^25^ Within the ≈7 µm thick PPy‐containing redox‐active layer, the NCFs serve as flexible binders that ensure that the PPy‐containing layer adheres tightly to the NCF layer. As already indicated, the redox‐active PPy‐containing layer, however, has several functions. The layer not only acts as a mechanical supporting layer for the NCF layer but also serves as an extension of the NCF separator layer via its porous structure, facilitating ion transport within the material.^24^ The latter feature is crucial to the function of the redox‐active separator, as the presence of a nonporous redox‐active layer would restrict mass transport in the batteries. This restriction means that the porous redox‐active layer functions similar to the conventional separator (that it has replaced) with respect to ion transport between the electrodes. Hence, this situation is significantly different compared to when a thinner conventional separator is combined with a thicker electrode to yield the same total thickness, because mass transport within the electrode would be significantly slower when the thickness of the electrode is increased. Consequently, the thickness of the redox‐active layer can be increased without affecting mass transport in the electrode in contact with the redox‐active layer. The porous structure of the redox‐active layer thus allows the capacities of both the redox‐active layer and the electrode in contact with the layer to be accessed simultaneously. Therefore, the PPy‐containing layer of the porous redox‐active separator acts as an additional cathode layer, although it is physically integrated with the separator to provide a flexible separator unit with the required mechanical strength. Moreover, the conducting PPy layer can act as upper current collector, which may be beneficial to the overall battery performance. It should also be noted that although the PPy within the redox‐active layer is electronically conducting, the risk of short‐circuits is still very low when the PPy‐containing layer is in contact with a cathode material. This effect can be explained by the fact that PPy is reduced to its electronically insulating state upon contact with the anode. This “automatic fuse effect,” which would clearly not be seen for a conventional electronic conducting material, enables the use of a thin NCF and a thicker redox‐active layer without increasing the risk of short‐circuits. This effect is in good agreement with the long‐time cycling results obtained with a symmetric Li/Li cell containing a PPy‐NCF membrane (Figure S3, Supporting Information) as well as by the cycling of a Li/LFP cell described in the section below.

### 2.2 The Pore Structure

**Figure 3:**
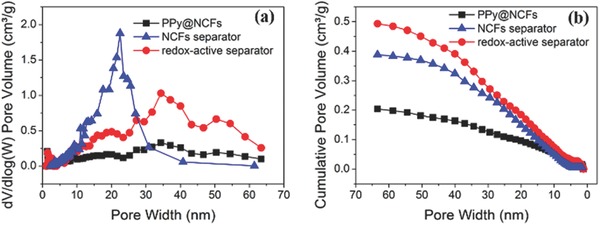
Pore structures of the different membranes. a) Pore size distribution and b) cumulative pore volume for the NCF‐based separator, redox‐active separator and PPy@NCFs composite membrane.

The SEM images (Figure 2) indicated differences between the morphologies of the redox‐active separator and the membrane composed only of NCFs, as the former appeared to be more porous. This morphology was confirmed by porosity determinations in which a membrane composed only of PPy@NCFs was included as a reference sample. The porosities for the NCFs and PPy@NCFs membranes, and the redox‐active separator, were 48%, 56%, and 59%, respectively, whereas the specific surface areas, obtained from Brunauer–Emmett–Teller (BET) nitrogen adsorption analyses (Figure S4, Supporting Information), were 103, 66, and 133 m^2^ g^−1^, respectively. The nitrogen adsorption isotherms were found to be very similar (i.e., of type IV) for all three samples, indicating the presence of both micro‐ and mesopores.^23^ This finding is also supported by the pore size distribution curves obtained with Barrett–Joyner–Halenda (BJH) nitrogen desorption (**Figure** **3**). We have previously shown that NCF‐based separators provide efficient ion transport and adequate insulation in electrochemical cells.^16^ The micro‐ and mesoporous structure of the redox‐active separator was almost identical to that of the PPy@NCF membrane, exhibiting BJH pore diameters in the range of 10–60 nm (and three peaks located at 20, 35, and 52 nm), while the NCFs membrane contained smaller pores of up to 40 nm (and a peak at 20 nm).

The pore size and total pore volume of the redox‐active separator were larger than those of the other two membranes, and the mesopore volume of the redox‐active separator was also larger than those of the NCF and PPy@NCF membranes. The large pore size, high porosity, and the well‐retained mesoporous structure are expected to be beneficial to LIB performance since these characteristics facilitate ion transport in LIBs.^26^, ^27^ The electrochemical impedance spectroscopy (EIS) results (Figure S5, Supporting Information) further indicated that the ionic conductivity of the redox‐active separator (i.e., 0.81 mS cm^−1^) was higher than those previously found for the NCF membrane (0.4 mS cm^−1^) and the polyethylene (PE) separator (0.13 mS cm^−1^).^16^ These results clearly demonstrate that the inclusion of PPy@NCFs results in separators with higher porosities and lower resistances compared to separators composed merely of NCFs.

### 2.3 Thermal Stability and Electrolyte Wettability

**Figure 4:**
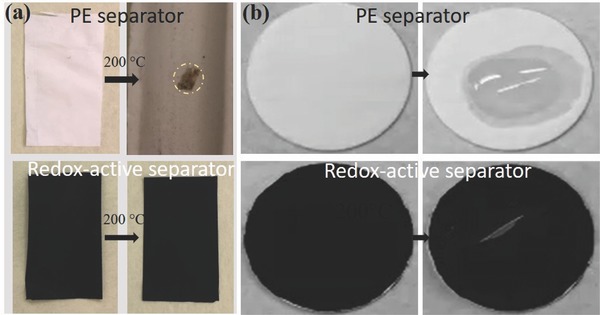
a) Thermal stability of the PE (upper photos) and redox‐active (lower photos) separators at elevated temperatures (the left and right photos were taken before and after heat treatment, respectively). b) Electrolyte wettability test of the PE and redox‐active separators (the left and right photos were taken before and after the addition of the electrolyte, respectively). The electrolyte spreading processes can be further compared in Video S1 in the Supporting Information.

Prior to exploring the electrochemical performance of the redox‐active separators in LIBs, we studied their thermal stability and electrolyte wettability. Commercial PE separators generally have a relatively low melting point (≈120 °C) and easily deform when exposed to temperatures above 100 °C. In contrast, the NCF‐based separators exhibited a good thermal stability,^16^ and it is also known that cellulose can maintain its chemical structure and original morphology at temperatures up to 300 °C.^28^ Measurements of the dimensional changes after subjecting the separators to 200 °C for 5 min indicated no visible dimensional change for the redox‐active separator, while the PE separator shrank by ≈94% immediately (**Figure** **4**a). This superior temperature tolerance of the redox‐active separators could effectively prevent internal short‐circuiting at evaluated temperatures.

The inherent hydrophilicity of the NCFs and the extended porous PPy@NCF structure also results in redox‐active separators with remarkable electrolyte wettability.^29^ Figure 4b; Video S1 in the Supporting Information demonstrates the electrolyte wettability differences between the redox‐active and PE separators, based on the spreading speed of the LP40 electrolyte. When LP40 was dropped onto the commercial PE separator, it remained as a droplet, whereas the electrolyte drops promptly diffused over the entire area of the redox‐active separator. The excellent thermal stability and wettability constitute important advantages of the redox‐active separators in terms of operational safety and assembly ease when used in LIBs.

### 2.4 Additional Capacity

**Figure 5:**
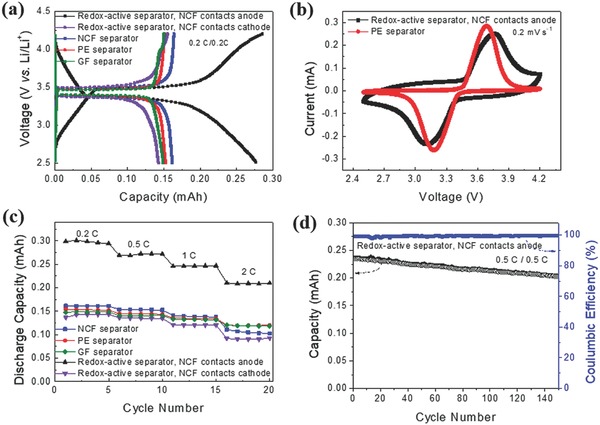
Battery performance for cells comprising LiFePO_4_ cathodes, Li anodes, and different separators. a) Charge/discharge profiles obtained at a rate of 0.2 C. b) Cyclic voltammograms recorded at a scan rate of 0.2 mV s^−1^. c) Discharge capacity as a function of the cycling rate. d) Capacity and Coulombic efficiency as a function of the cycle number for Cell I. The thicknesses of the redox‐active, NCF, PE, and GF separators were 10, 10, 25, and 255 µm, respectively.

To investigate the capacity‐providing function of the redox‐active separator, proof‐of‐concept cells comprising the Li metal anodes and LFP cathodes were assembled. In one cell type (denoted Cell I), the PPy‐containing layer of the redox‐active separator contacted the LFP cathode. In the other cell type (denoted Cell II), the PPy‐containing layer instead contacted the Li metal anode. For comparison, LFP half cells with NCFs, PE, and glass fiber (GF) separators were also assembled. **Figure** **5**a shows the charge/discharge voltage profiles of the LFP cells equipped with the different separators obtained at a rate of 0.2 C. The highly reproducible and well‐defined plateaus at ≈3.4 V, which are characteristic of the LFP electrodes, demonstrate that the redox‐active separator does indeed function well as an LIB separator. Compared to the cells containing the other separators, Cell I (with the PPy‐containing layer contacting the LFP cathode), exhibited a longer plateau and a sloping region stemming from the PPy redox capacity. The discharge capacity of Cell I (0.276 mA h) was therefore higher than those obtained with the NCF separator (0.16 mA h), the PE separator (0.152 mA h), and the GF separator (0.15 mA h), even though the LFP electrodes in these cells all had similar weights. Cell II, on the other hand, showed a capacity of 0.142 mA h, which was comparable to those obtained with the PE or GF separators, indicating that there was no capacity contribution from the PPy‐containing layer when in contact with the Li electrode. The latter is, however, not surprising as the capacity of this half‐cell was controlled by the LFP electrode rather than the Li electrode. As already mentioned, the PPy in contact with the Li electrode would also be reduced to its electronically insulating state, whereas the PPy should remain electronically conducting and redox‐active within the LFP potential window.

These results thus confirm that the highly porous, redox‐active PPy‐containing layer functions as a combined separator and porous electrode and the PPy capacity merely was added to that of the LFP in Cell I. It should also be mentioned that the iR drop within the electrolyte inside the PPy‐containing layer can be expected to be small.^30^ In Figure 5c, the results show that Cell I exhibited higher capacities than those obtained for the cells equipped with the NCF or PE separators at all rates (i.e., 0.2–2 C) and Cell I still delivered a capacity of 0.203 mA h at 2 C. Note that the LIB containing the redox‐active separator exhibited a lower rate capability than those of the LIB with the PE separator, due to the low rate capability of PPy‐containing layer employed in this proof‐of‐concept study. As the risk of PPy overoxidation (yielding a lower PPy capacity) increases as the upper cut‐off voltage is increased, care should also be taken when employing the present redox‐active separators in conjunction with high‐voltage cathode materials. No indications of short‐circuits were observed during the cycling of the cells containing the redox‐active separators, and a capacity of 0.2 mA h could still be obtained after 150 cycles at a rate of 0.5 C (Figure 5d). This finding is in good agreement with previously reported results demonstrating that thin mesoporous cellulose layers can be used as effective separators in LIBs.^20^

**Figure 6:**
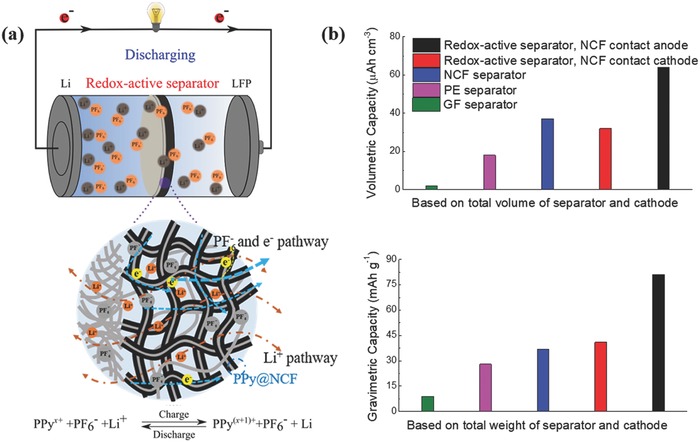
a) Schematic description of the LFP/Li batteries equipped with redox‐active separator with NCFs in contact with the Li anode. b) Gravimetric/volumetric capacity as a function of the weight/volume of various separators and cathode for the different LFP/Li cells.

The higher capacity of Cell I can thus be attributed to the additional capacity provided by the oxidation and reduction of PPy, i.e., $[\mathrm{PPy}]\;+\;{\mathrm{PF}}_{6}^{-}\;↔\;[{\mathrm{PPy}}^{+}]{\mathrm{PF}}_{6}^{-}\;+\;e^{-}$.^31^ These reactions, which typically are associated with the insertion and extraction of anions (rather than Li^+^ intercalation and deintercalation as is the case for the LFP electrode) also explain the sloping regions in the charge/discharge curves (Figure 5a) as well as the rather broad peaks at ≈3.15 and 3.75 V seen in the cyclic voltammograms (Figure 5b). The PPy capacity contribution was assessed in an experiment, in which the redox‐active separator was used as a cathode in a cell not containing any other cathode. The charge/discharge curves of the redox‐active separator were relatively linear and symmetrical (Figure S6, Supporting Information), in good agreement with previous findings for PPy/Li batteries.^31^ As the discharge capacity of the redox‐active separator was ≈0.085 mA h (i.e., 84 mA h g^−1^ based on the PPy mass, which corresponds to approximately 80% of the theoretical capacity of PPy), it is immediately evident that the redox‐active separator functioned both as a separator and as a cathode in this cell. These results clearly show that the redox‐active separator containing PPy can be used to increase the capacity of the batteries if the capacity of the battery is controlled by the capacity of the cathode. Although the capacity contribution from PPy strongly depends on the active mass of the cathode, it should be mentioned that the mass of PPy in the redox‐active layer can also be increased further. It is therefore reasonable to conclude that the redox‐active separator concept is feasible and of further interest to the development of functional LIB separators. **Figure** **6**a shows a conceptual illustration depicting the different functionalities of the redox‐active separator toward the enhanced capacity for LIBs.

### 2.5 Enhanced Volumetric/Gravimetric Capacity

The LFP/Li cells equipped with different separators, but identical LFP cathodes, were used to investigate the influence of the separator type on the LIB capacity. One important point here is that a thicker separator results in a larger cell weight/volume (Table S2, Supporting Information), which gives rise to lower volumetric capacities (i.e., energy densities). As the thickness of the separator is increased, the volumetric performance thus decreases (Figure 6b). In the proof‐of‐concept study, a volumetric capacity of only 1.8 µAh cm^−3^ was obtained for the cell with the 255 µm thick GF separator after normalization with respect to the total volume of the separator and cathode. This value should be compared with the capacity of 18 µAh cm^−3^ found with the conventional 25 µm PE separator. The LIBs equipped with the thinner separators (i.e., the 10 µm redox‐active separator) consequently exhibited higher volumetric capacities as a volumetric capacity of 34 µAh cm^−3^ was found for Cell II, while a value of 67 µAh cm^−3^ was obtained for Cell I, in which the redox capacity of PPy was also exploited. By making a part of the separator redox‐active, it is hence possible to make better use of the volume between the electrodes, especially if the redox‐active part also provides mechanical support to the NCF layer. The flexibility of the redox‐active separator and the inherent “automatic fuse effect” of the redox‐active layer are clearly also very important for safety reasons.

The results also demonstrate that higher gravimetric capacities (after normalization with respect to the total weight of the separator and cathode) can be reached with the redox‐active separator (Table S2, Supporting Information) due to the additional capacity stemming from PPy. In this comparison, the electrolyte weight was not taken into account since the same amount of electrolyte was used in all the cells. As shown in Figure 6b, a discharge capacity of 81 mA h g^−1^ could be obtained for Cell I, while the corresponding values for the cells with the PE and GF separators were 26 and 8 mA h g^−1^, respectively. One can also compare the gravimetric capacities based on the LFP mass, as is shown in Figure S7 in the Supporting Information. For Cell I (with the redox‐active separator in contact with the LFP electrode), a capacity of 257 mA h g^−1^ was obtained due to the additional capacity provided by PPy in the redox‐active separator. The results consequently demonstrate that the redox‐active separator approach may be used to increase the volumetric and gravimetric capacities of LIBs. The approach is particularly interesting in cases where the capacity of a battery needs to be increased without changing the electrode materials or the volume of the cell.

In the redox‐active separator, the exploitation of the PPy redox capacity is significantly facilitated by the fact that the PPy‐containing composite is highly porous and the PPy coating on the cellulose fibers is merely approximately 50 nm thick.^28^ This facilitation means that the present approach is significantly different from other approaches in which the PPy has been included in the cathode. While it has been shown that the capacity and rate capability of LFP cathodes may be improved by the addition of redox‐active polymer coatings,^32^, ^33^ these improvements have generally been attributed to an increased electrode conductivity due to the presence of the electronically conducting polymer (which most likely also served as an additional binder in many cases). To date, conducting polymers such as PPy or polyaniline (PANI) have mainly been employed in conjunction with cathode engineering and only in cells containing conventional separators. Since relatively small amounts of redox‐active polymers generally were used, the obtained capacity increases were also relatively small. It should, however, be mentioned that PPy‐modified separators have also been used to improve the electrochemical performance of Li–sulfur batteries.^34^, ^35^ In these cases, however, the improvement can mainly be ascribed to the interactions between PPy and the lithium polysulfides, and the PPy conductivity,^36^ as the capacity contribution due to the PPy redox reactions, should be practically negligible due to the low capacity of PPy within the sulfur cathode working potential window (i.e., 1.8–2.8 V). The use of PPy‐modified separators is therefore unlikely to lead to increased volumetric/gravimetric capacities for Li–S batteries. In the present case, PPy is instead used as an integrated part of the cellulose‐based separator in the form of a porous PPy@NCF composite. One important advantage of this approach is that the porous structure of the composite allows larger amounts of PPy to be used, which naturally provides larger increases in the total cathode capacity. As the PPy redox reactions involve the uptake and release of anions, while Li^+^ ions are released and inserted during the oxidation and reduction of LFP, the two cathode reactions are complementary. The influences of the PPy redox reactions on the oxidation and reduction of LFP should hence be small.

To further demonstrate the advantages of the redox‐active separator, the electrochemical performance of Cell I was compared with that of a cell comprising a LFP‐PPy composite cathode and a Li anode. The LFP‐PPy cathode was prepared using a slurry containing LFP and PPy@NCFs, and the PPy weight ratio was the same as that of the redox‐active separator (see the Experimental Section). From the SEM images in Figure S8 in the Supporting Information, the LFP and PPy@NCFs were uniformly distributed in the electrode and the LFP fraction was lower for the LFP‐PPy electrode than that of the LFP electrode employed in Cell I. Based on our measurements, a thickness of 7 µm was required to obtain an LFP mass loading of 1 mg cm^−2^ for the LFP electrode used in Cell I, while the corresponding thickness for the LFP‐PPy electrode was 41 µm. Hence, the LFP‐PPy electrode had to be constructed almost six times as thick to have the same capacity as the LFP electrode used in conjunction with the PPy‐containing redox‐active separator. Such an increased LFP‐PPy electrode thickness will decrease the volumetric power density attainable for an LIB significantly.^37^ Hence, these results demonstrate that the inclusion of any redox‐active component (e.g., PPy) into the LFP cathode itself is an inefficient way to increase the capacity of the cathode compared to the redox‐active separator approach.

**Figure 7:**
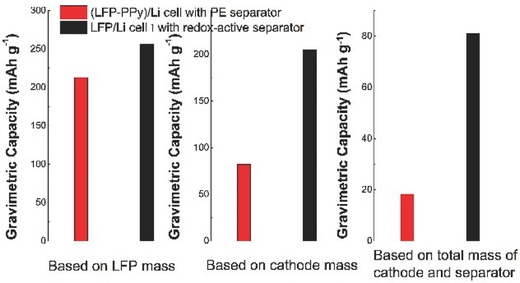
Comparison of the gravimetric capacities of a (LFP‐PPy)/Li cell equipped with a PE separator and Cell I that contains a redox‐active separator.

Interestingly, note that the charge/discharge profiles for the LFP‐PPy‐based Li cell featured a long plateau region and a linear sloping region due to the LFP and PPy redox capacities, respectively, which is analogous with the results for Cell I. However, as demonstrated in Figure S9 in the Supporting Information, the rate capability of the LFP‐PPy cathode‐based cell was poor, and the discharge capacity obtained at 0.2 C was also lower compared to that of Cell I (i.e., 213 vs 257 mA h g^−1^) when normalized with respect to the LFP mass (note that the PPy weight ratios were the same in both cases). A comparison of the capacities for the LFP‐PPy‐based cell and Cell I after normalization with respect to the total weight of the cathode (including the weight of the active materials in the cathode, carbon black and binder) is presented in **Figure** **7**. Based on the total weight of the cathode, discharge capacities of 205 and 82 mA h g^−1^ were achieved for Cell I and the LFP‐PPy‐based cell, respectively. If the total weights of the cathode and separator were considered, Cell I still exhibited a higher capacity than that of the LFP‐PPy‐based cell (i.e., 81 vs 18 mA h g^−1^). It is therefore much more efficient to employ PPy in a redox‐active separator than to include it directly in the LFP cathode.

Based on the results described above, it is immediately evident that redox‐active separators, comprising even thicker PPy‐containing layers, should provide even higher capacities. To demonstrate this, LFP/Li cells with thicker redox‐active separators were assembled and tested in the Cell I configuration. As shown in Figure S10 in the Supporting Information, a capacity of 0.325 mA h could then be obtained at 0.2 C using a 17 µm thick redox‐active separator comprising a 14 µm thick PPy‐containing layer.

When using a commercial cathode with a larger mass loading and a higher thickness (i.e., ≈10 mg cm^−2^ and 60 µm, respectively), the cell capacity increases because the redox‐active separator is naturally significantly smaller than that described above. To investigate this effect, a 17 µm thick redox‐active separator was employed together with an LFP cathode with a mass loading of ≈5.5 mg cm^−2^. As shown in Figure S11 in the Supporting Information, this LFP/Li cell exhibited a capacity of 161 mA h g^−1^ at 0.2 C, while a capacity of 143 mA h g^−1^ was obtained with a corresponding cell containing a PE separator. Since the separator thickness in a commercial cell is 25 or even 40 µm compared with the 17 µm used in the present proof‐of‐concept cells and the thickness of the redox‐active separator can be readily increased, it is reasonable to assume that noticeable capacity increases and should be observed even for cells containing cathodes with mass loadings more similar to those of commercial cells. To develop a redox‐active separator for practical applications requiring higher additional capacities, further studies would clearly be needed to optimize the electroactive material in the redox‐active separators while maintaining their porous structure and all the other properties that are required of a well‐functioning LIB separator. One possible approach is to further increase the PPy capacity, since capacities up to 120 mA h g^−1^ (compared to 84 mA h g^−1^ here) have been reported.^38^ It is also be possible to use redox‐active separators based on other conducting polymers such as PANI, for which a stable capacity of 230 mA h g^−1^ has been reported within similar working potential windows.^39^

## 3 Conclusions

The results have demonstrated that the present proof‐of‐concept redox‐active separator, which contains a mesoporous insulating NCF layer and a redox‐active PPy‐containing support layer, can be used to enhance the capacity of LIBs. The flexible redox‐active separator can readily be manufactured employing a straightforward paper‐making process, and the porous structure of the redox‐active separators yielded an ionic conductivity of ≈0.8 mS cm^−1^. The results indicate that ≈3 µm thin insulating NCF layer was sufficient to prevent short‐circuits in the LIBs and PPy was reduced into an electronically insulating form if the PPy layer encountered a lithium electrode. This finding suggests that the NCF layer can be made as thin as ≈3 µm without jeopardizing cell safety. By replacing a conventional separator with the redox‐active separator, the capacity of a proof‐of‐concept battery comprising a LiFePO_4_ cathode and a Li metal anode could be increased from 0.16 to 0.276 mA h. Based on the total volume (or weight) of the separator and cathode, the capacity could be increased from 18 µAh cm^−3^ (or 26 mA h g^−1^) to 67 µAh cm^−3^ (or 81 mA h g^−1^). Although the actual capacity contribution due to the redox‐active separator depends on the PPy mass loading in the redox‐active separator and that of the cathode, the results obtained with the proof‐of‐concept cells clearly indicate that the redox‐active separator concept holds considerable promise for use in high energy density thin‐film LIBs, and possibly also other electric energy storage devices.

## 4 Experimental Section

*Materials*: The nanocellulose fibers were obtained from FMC Biopolymers (USA) while iron(III) chloride hexahydrate, sodium chloride, hydrochloric acid, pyrrole, and Tween‐80 were purchased from Sigma‐Aldrich and used without any further purification. The Solupor separator, LP40 (i.e., 1.0 m lithium hexafluorophosphate (LiPF_6_) in ethylene carbonate/diethyl carbonate (1/1, v/v), BASF), the nylon filter membrane (ø90 mm, 0.45 µm; Magna), and the Li foil (125 µm; Cyprus Foote Minerals) were purchased from the indicated suppliers and used as received. The other materials (e.g. LFP, deionized water, ethanol, Al foil, and Cu foil) were also bought from commercial manufacturers and used as received.

*Preparation of the PPy@NCFs Composites*: The NCFs (300 mg) were first dispersed in 60 mL water using sonication with a total pulse time of 10 min and water cooling. Pyrrole (1.0 mL) and 0.5 m HCl (60 mL) were mixed with the NCFs dispersion using magnetic stirring for 5 min. PPy was then formed on the NCFs via chemical polymerization employing FeCl_3_·6H_2_O (8.7 g) dissolved in 0.5 m HCl (60 mL) as the oxidant. The polymerization was allowed to proceed for 30 min under stirring, after which the composite was collected in a Büchner funnel connected to a suction flask and washed with 0.5 m HCl (3 L) followed by deionized water (1 L). The washed composites were then drained on a polypropylene filter to form filter cakes, which subsequently were collected and dispersed in 200 mL water by sonication to form a uniform PPy@NCFs suspension. The PPy weight fraction in the composites was, as previously demonstrated,^23^, ^28^ about 70%.

*Preparation of the Redox‐Active Separators*: The preparation of the redox‐active separators is schematically shown in Figure 2a. To obtain a uniform NCF dispersion, 8 mg of NCFs powder was first dispersed in 60 mL water by sonication. This dispersion was then vacuum filtered through a nylon membrane to generate the bottom NCFs layer. Subsequently, another 60 mL dispersion containing 2 mg of NCFs and varying amounts of the PPy@NCFs composite was then poured onto the surface of the NCFs layer under vacuum filtration to form a filter cake. The latter was subsequently dried to form a soft flexible paper sheet. By adjusting the amounts of PPy@NCFs in the suspension, electroactive separators with total thicknesses of 10 and 17 µm were obtained (the thickness of the NCF layer was about 3 µm in both cases), containing about 0.34 and 0.65 mg cm^−2^ PPy, respectively. Since the separators were finally punched into circular sheets with a diameter of 2 cm, the actual amounts of PPy in the separators were ≈1.1 and 2.0 mg, respectively. A pure NCFs membrane with a thickness of 10 µm (used as a control sample) was also made using an analogous procedure.

*Structural Characterization and Analysis*: SEM images of all the samples were obtained with a Leo Gemini 1550 FEG SEM instrument (Zeiss, Germany) while nitrogen gas adsorption and desorption isotherms were recorded with an ASAP 2020 instrument (Micromeritics, USA). The specific surface area was calculated according to the BET method using the adsorption data, whereas the pore size distribution was determined using the BJH method. The apparent (true) density (ρ_a_) was measured by helium pycnometry (AccuPyc 1340, Micro‐meritics, USA), while the bulk density (ρ_b_) was calculated from the bulk mass and bulk volume of the separator. The porosity (*P*) was estimated based on the equation: *P* = (1−ρ_b_/ρ_a_) × 100% while the degree of thermal shrinkage was evaluated by measuring changes in separator (area‐based) dimensions after exposure to 200 ˚C for 5 min. The electrolyte wettability of the separators was assessed by measuring the electrolyte (i.e., LP40) spread speed on the materials.

*Electrochemical Characterization and Analysis*: LFP electrodes composed of LFP, carbon black, and polyvinylidene fluoride (PVDF) binder in a ratio of 8:1:1 by weight, were prepared by casting the obtained slurry onto Al foil. For comparison, LFP‐PPy cathodes were also fabricated from a mixture of the active materials (i.e., LFP and PPy@NCFs), carbon black, and PVDF (80:10:10 wt%), in which the LFP:PPy weight ratio was equal to the PPy:LFP weight ratio calculated based on the PPy amount in the redox‐active separator and the weight of the LFP used in the cathode. The electrodes were then dried at 80 °C overnight in a vacuum oven. The cathode sheet was subsequently punched into circular sheets with a diameter of 13 mm and a typical active mass loading of 1 mg cm^−2^, although electrodes with a mass loading of ≈5.5 mg cm^−2^ were also prepared. Two‐electrode pouch cells were made by sandwiching the electrolyte‐soaked separator between the LFP electrode and the Li foil counter electrode prior to sealing the cells in an argon‐filled glovebox (H_2_O content < 1 ppm, O_2_ content < 1 ppm). Note that, two different cell configurations were used with the redox‐active separator, one in which the NCF contacted the LFP cathode and one in which the NCF contacted the Li anode. The charge/discharge tests were performed between 2.5 and 4.2 V versus Li^+^/Li with a Arbin cycler (model BT‐2043) system at room temperature using different cycling rates (i.e., 0.2–2 C). The cycling rate was calculated based on the theoretical specific capacity of LFP. The thickness of the redox‐active separator was 10 µm, if not stated otherwise. The cyclic voltammetry experiments were carried out with a VMP instrument (Biologic Multichannel Potentiostat) between 2.5 and 4.2 V versus Li^+^/Li using various scan rates, whereas the EIS measurements were performed at a cell potential of 0 V, using an ac amplitude of 10 mV and frequencies between 100 kHz and 10 mHz.

## Conflict of Interest

The authors declare no conflict of interest.

## Supporting information

Supplementary

Supplementary
