# Supplementary material for: Redox‐Active Separators for Lithium‐Ion Batteries
Source: Adv Sci (Weinh). 2017 Dec 19;5(3):1700663. doi: 10.1002/advs.201700663 (PMC5867047; doi:10.1002/advs.201700663)
Supplement: Supplementary file 1 — Supplementary [file ADVS-5-1700663-s002.pdf]

Copyright WILEY-VCH Verlag GmbH & Co. KGaA, 69469 Weinheim, Germany, 2017.

ADVANCED SCIENCE
Open Access

Supporting Information

for *Adv. Sci.,* DOI: 10.1002/advs.201700663

Redox-Active Separators for Lithium-Ion Batteries

Zhaohui Wang, Ruijun Pan, Changqing Ruan, Kristina Edström, Maria Strømme, and Leif Nyholm**

SUPPLEMENTARY INFORMATION

Redox-active Separators for Lithium-ion Batteries

Zhaohui Wang, Ruijun Pan, Changqing Ruan, Kristina Edström, Maria Strømme, Leif Nyholm**

Dr. Z.H. Wang, R.J. Pan, Prof. K. Edström, Prof. L. Nyholm

Department of Chemistry-The Ångström Laboratory, Uppsala University, Box 538, SE-751 21 Uppsala, Sweden

E-mail: zhaohui.wang@kemi.uu.se; Leif.Nyholm@kemi.uu.se

C.Q. Ruan, Prof. M. Strømme

Nanotechnology and Functional Materials, Department of Engineering Sciences, The Ångström Laboratory, Uppsala University, Box 534, SE-751 21 Uppsala, Sweden

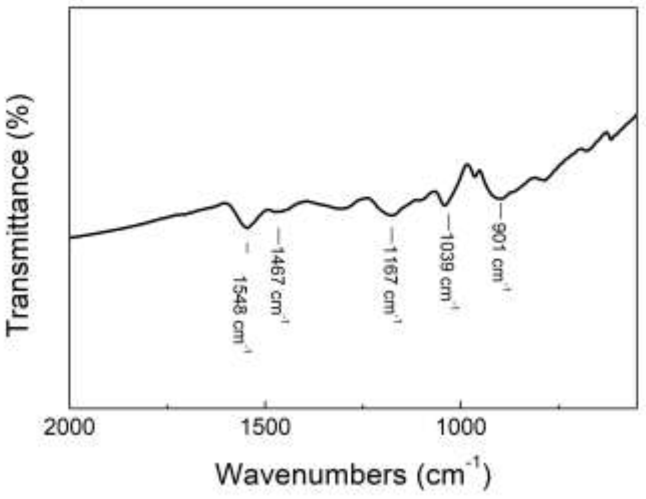

Figure S1. FTIR spectrum for the PPy@NCFs composite.

The characteristic peak at 901 cm^{-1} is related to the C-H out-plane vibration, whereas the 1167 cm^{-1} band can be assigned to a C-N-C stretching vibration in the polaron structure of pyrrole rings. The bands at 1039 and 1300 cm^{-1} can be ascribed to C-H in-plane vibration and in-plane N-H deformation, respectively. The characteristic bands at 1466 and 1548 cm^{-1} are attributed to the C=C and C-N stretching deformation mode of the pyrrole rings.[1, 2] This FTIR spectrum thus demonstrates that PPy was indeed present in the composites.

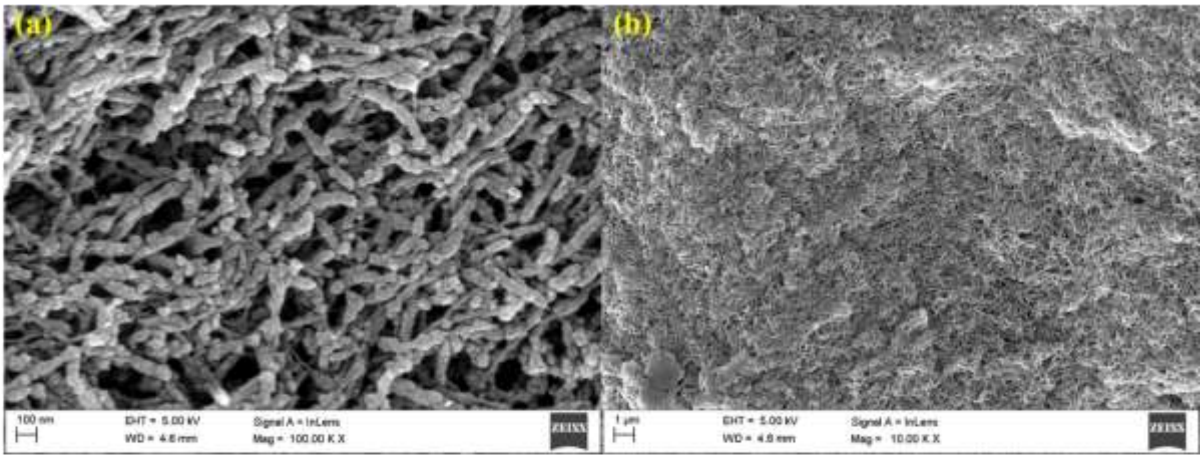

Figure S2. Scanning electron microscopy (SEM) images for PPy@NCFs composites at (a) high and (b) low magnification.

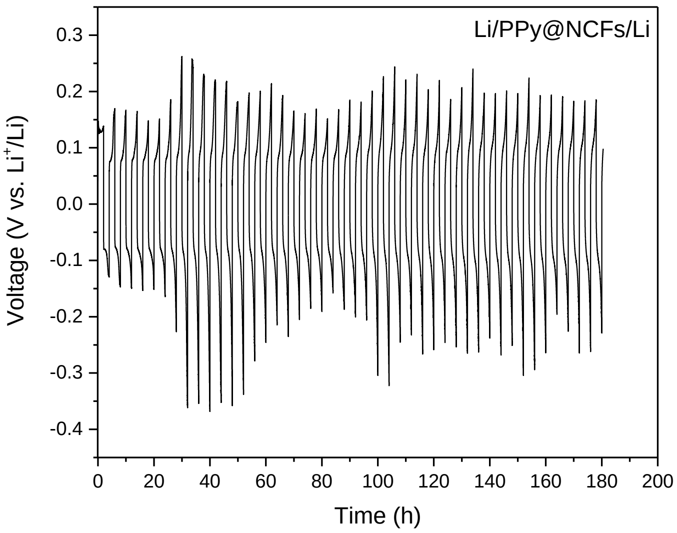

Figure S3. Cycling curve of a Li/Li symmetric cell with PPy@NCFs membrane as the separator (1 mA, 2h cycling).

Due to the low electrochemical potential of Li metal, the conductive PPy was reduced to its insulating state and the Li/Li symmetric cell could therefore operate with a PPy@NCFs membrane without any short-circuits.

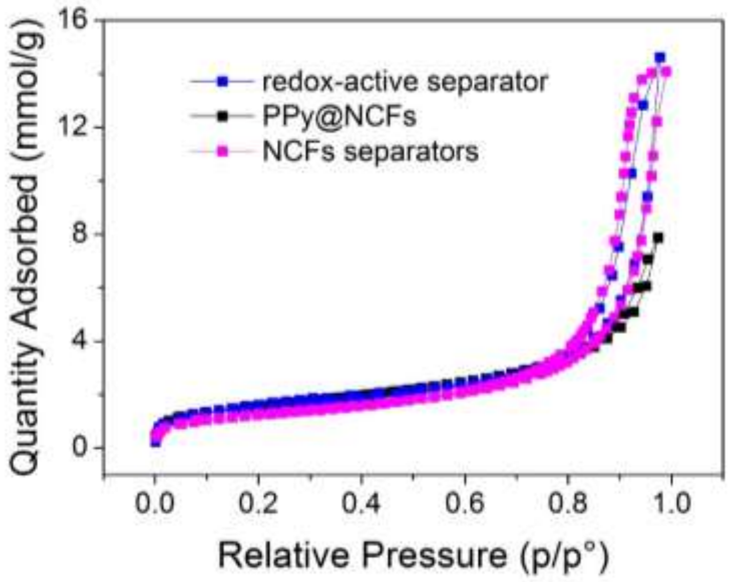

Figure S4. Nitrogen adsorption/desorption isotherms for the nanocellulose fibers (NCFs), PPy@NCFs and redox-active separators.

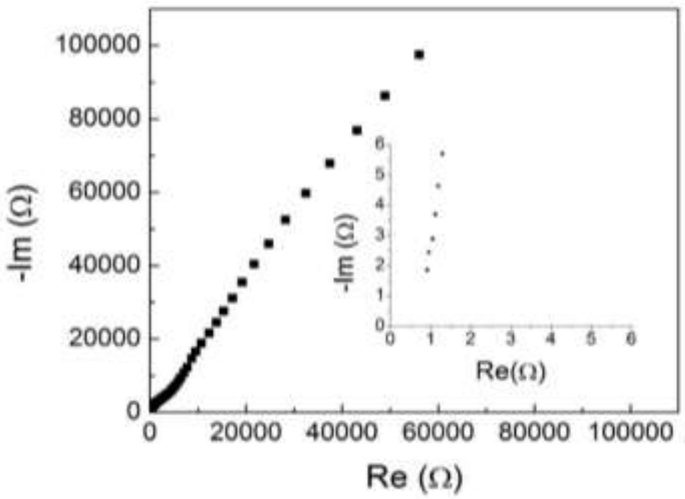

Figure S5. Nyquist plots of electrochemical impedance spectroscopy measurements on a cell composed of two stainless steel electrodes separated by a redox-active separator; the inset shows a magnification of the high frequency region.

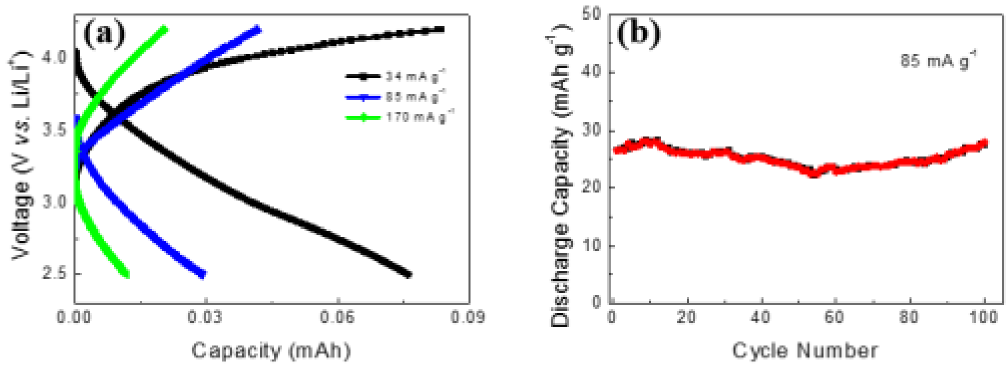

Figure S6. Electrochemical performance of the redox-active separators: (a) charge/discharge curves recorded at various current densities; the electrochemical behavior of the redox-active separator studied in a cell with a Li metal anode facing the NCFs layer and an Al current collector facing the PPy-containing layer. (b) The discharge capacity as a function of the cycle number. Note that 34 mA g^{-1} corresponds to 0.2 C for the LFP cathode shown in Figure 5a and that the rate of discharge and charge thus was higher in Figure S6b than in Figure 5 in the main text.

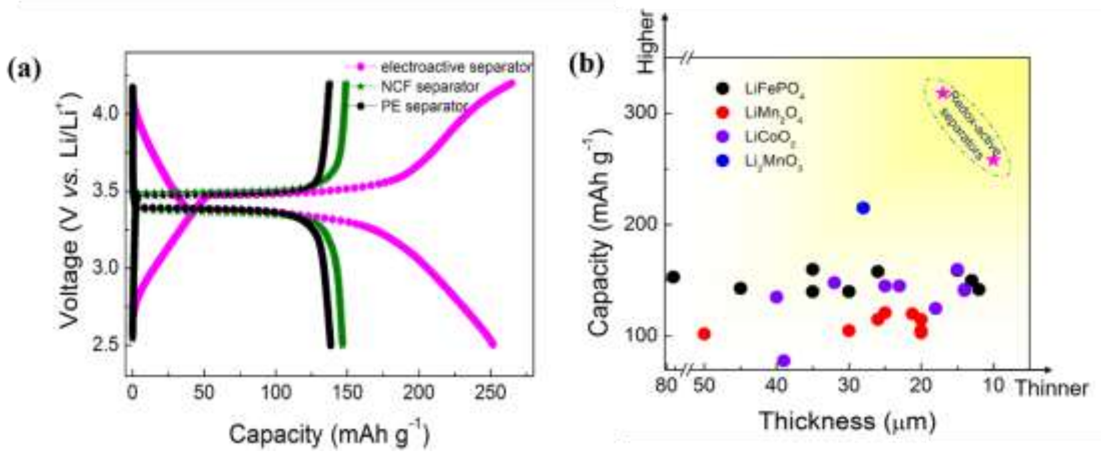

Figure S7. Performance for LFP/Li cells equipped with different separators. (a) Comparison of the charge/discharge profiles obtained at a rate of 0.2 C. (b) Active mass normalized capacities for LIBs containing various cathodes as a function of the separator thickness; see **Table S1** for details.

After normalization with respect to the LFP mass, a discharge capacity of 257 mAh g^{-1} was obtained for the cell containing the redox-active separator due to the PPy capacity contribution. This value is much higher than those obtained with the NCF and PE separators (150–160 mAh g^{-1}) as well as with many other commercial and synthetic separators used in LFP based cells or other types of LIBs (120-160 mAh g^{-1}) (see Table S1). It can be also be concluded that the PPy capacity was about 100 mAh g^{-1} and that this value was higher than the 84 mAh g^{-1} obtained in the Li/PPy cell. This is, however, not surprising since the charge/discharge rate was lower in the latter case (see Figure S6).

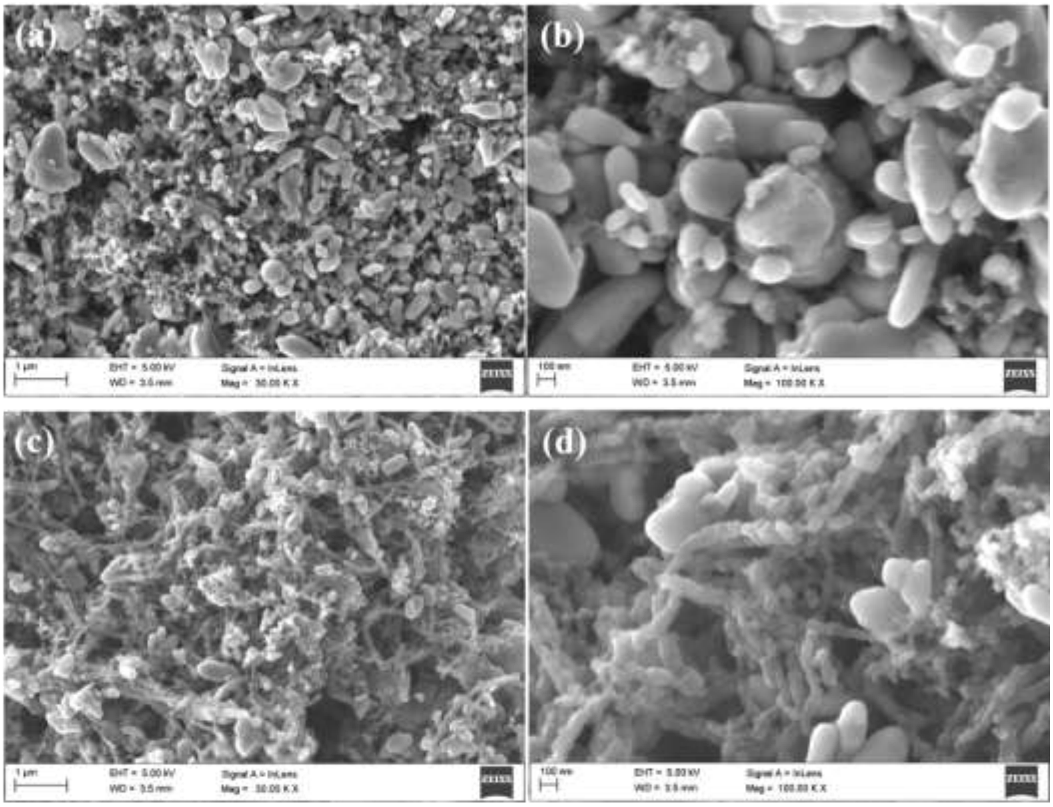

Figure S8. SEM images recorded at different magnifications for a (a, b) LFP cathode and (c, d) a LFP-PPy cathode.

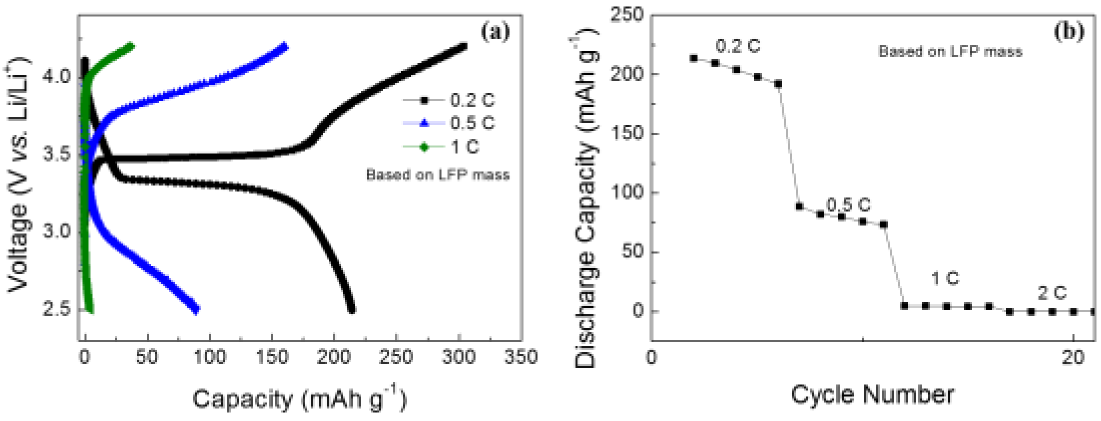

Figure S9. Battery performance for the LFP-PPy cathode when used together with a PE separator: (a) charge/discharge curves recorded at various cycling rates; (b) the discharge capacity as a function of the rate and cycle number. The poor rate capability for the LFP-PPy cathode was most likely due to the mass transport limitations within the much thicker cathode, and the lower conductivity with insufficient conductive network of the PPy matrix in the LFP-PPy cathode as compared to carbon black in the LFP cathode (also can be deduced from Figure S6).

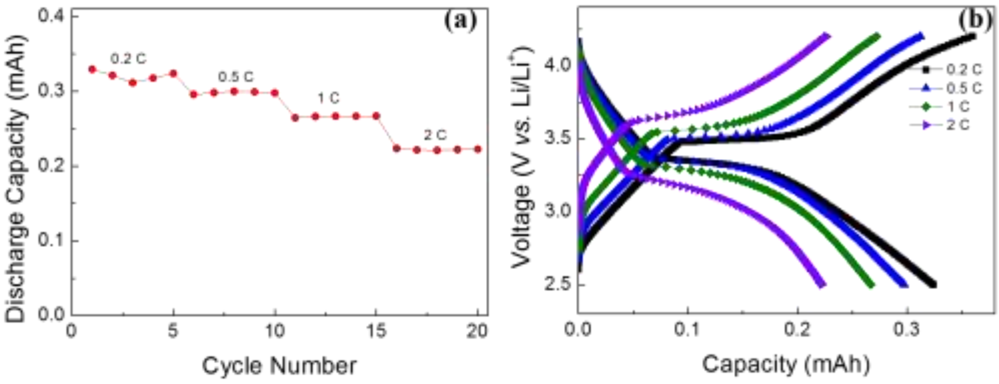

Figure S10. Electrochemical performance of the LFP cathode in a cell containing a 17 μm-thick redox-active separator: (a) discharge capacity as a function of the cycling rate and cycle number; (b) charge/discharge curves recorded at different cycling rates.

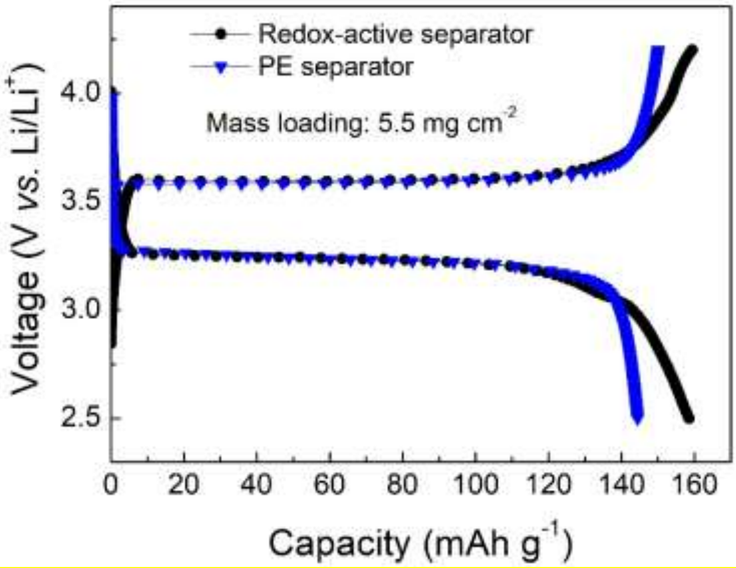

Figure S11. Comparison of the charge/discharge profiles obtained at a rate of 0.2 C for LFP/Li cells equipped with high mass loading cathodes and different separators.

Table S1. Overview of the properties of separators used in LIBs. The highest value from each reference is reported.

Separators	Thickness	Porosity	Cathode (Capacity)	Ref.
cellulose/PDA	40 μm	62%	$LiCoO_2$ (135 mAh g^{-1})	[3]
nanocellulose	35 μm	46%	$LiFePO_4$ (140 mAh g^{-1})	[4]
Nf-PP-Li	21.2 μm	/	$LiMn_2O_4$ (120 mAh g^{-1})	[5]
PE	12 μm	20%	$LiFePO_4$ (142 mAh g^{-1})	[4]
PE-SiO_2@PDA	26 μm	44%	$LiMn_2O_4$ (115 mAh g^{-1})	[6]
PE-SiO_2	26 μm	47%	$LiMn_2O_4$ (115 mAh g^{-1})	[6]
PE	20 μm	44%	$LiMn_2O_4$ (115 mAh g^{-1})	[6]
PE-alt-$MALi_2$	50 μm	20%	$LiMn_2O_4$ (102 mAh g^{-1})	[7]
ZrO_2/POSS-PE	14 μm	47%	$LiCoO_2$ (141 mAh g^{-1})	[8]
PEGDA-PEI/PVDF	35 μm	64%	$LiFePO_4$ (160 mAh g^{-1})	[9]
CDA-SiO_2-PE	15 μm	40%	$LiCoO_2$ (160 mAh g^{-1})	[10]
CNF/PVP/PAN	20 μm	65%	$LiMn_2O_4$ (105 mAh g^{-1})	[11]
PP/PE/PP	20 μm	40%	$LiMn_2O_4$ (103 mAh g^{-1})	[11]
PVA/CNF	23 μm	65%	$LiCoO_2$ (145 mAh g^{-1})	[12]
Al_2O_3:LPMA64/PP	26 μm	/	$LiFePO_4$ (158 mAh g^{-1})	[13]
WCDA-SiO_2-PE	18 μm	/	$LiCoO_2$ (125 mAh g^{-1})	[14]
F-silica/PVP/PAN	30 μm	54%	$LiMn_2O_4$ (105 mAh g^{-1})	[15]
SENS	76 μm	/	$LiFePO_4$ (153 mAh g^{-1})	[16]
(MWNT/PEI)/ F-silica/PVP/PAN	28 μm	/	Li_2MnO_3 (215 mAh g^{-1})	[17]
PDA/PE	25 μm	40%	$LiMn_2O_4$ (121 mAh g^{-1})	[18]
PEO/PE	25 μm	40%	$LiCoO_2$ (145 mAh g^{-1})	[19]
PI	15 μm	65%	$LiFePO_4$ (159 mAh g^{-1})	[20]
$MgAl_2O_4$/PVDF-HFP	30 μm	60%	$LiFePO_4$ (140 mAh g^{-1})	[21]
Bacterial CNF	13 μm	/	$LiFePO_4$ (150 mAh g^{-1})	[22]
PEI-SiO_2/PE	14 μm	47.6%	$LiCoO_2$ (142 mAh g^{-1})	[23]
LLZO/PVDF-HFP	32 μm	39%	$LiCoO_2$ (-148 mAh g^{-1})	[24]
Cellulose	39-85 μm	/	$LiCoO_2$ (78 mAh g^{-1})	[25]

Rice paper	100 μm	/	$LiFePO_4$ (158 mAh g^{-1})	[26]
Glass fiber/PI	45 μm	73%	$LiFePO_4$ (143 mAh g^{-1})	[27]
redox-active separator	10 μm	60%	$LiFePO_4$ (257 mAh g^{-1})	this work
redox-active separator	17 μm	65%	$LiFePO_4$ (320 mAh g^{-1})	this work

Abbreviations and specialized terms used in Table S1.

PDA: Polydopamine; PP: Polypropylene; PE: polyethylene; PVDF: polyvinylidene fluoride; PVDF-HFP: polyvinylidene fluoride-hexafluoropropylene; POSS: Polyhedral oligomeric silsesquioxane; PEGDA: poly(ethylene glycol diacrylate); PEO: poly(oxyethylene); PVA: polyvinyl alcohol; PEI: Polyethylenimine; PVP: poly(vinylpyrrolidone); PAN: polyacrylonitrile; CNF: cellulose nanofiber; Nf-PP: Nafion coated Polypropylene; SENS: Silica Encapsulated Nanofibrous Separator; F-silica: thiol (-SH)-functionalized silica; WCDA: water-based cellulose diacetate; LLZO: garnet-type fast lithium ion conductor $Li_7La_3Zr_2O_{12}$; Li_2MnO_3: $0.49Li_2MnO_3{\cdot}0.51LiNi_{0.37}Co_{0.24}Mn_{0.39}O_2$

Table S2. Weight and volume of the separators used in this study.

Separator	weight	thickness	Volume
Redox-active separator	2.05 mg	10 μm	0.0031 cm^{-3}
NCF separator	3.06 mg	10 μm	0.0031 cm^{-3}
PE separator	4.6 mg	25 μm	0.0078 cm^{-3}
GF separator	16.4 mg	255 μm	0.0790 cm^{-3}

References

[1] N. Su, H. Li, S. Yuan, S. Yi, E. Yin, *Express Polym Lett.* **2012**, *6*, 697.

[2] J. Tabačiarová, M. Mičušík, P. Fedorko, M. Omastová, *Poly. Degrad. Stability* **2015**, *120*, 392.

[3] Q. Xu, Q. Kong, Z. Liu, J. Zhang, X. Wang, R. Liu, L. Yue, G. Cui, *RSC Adv.* **2014**, *4*, 7845.

[4] R. Pan, O. Cheung, Z. Wang, P. Tammela, J. Huo, J. Lindh, K. Edström, M. Strømme, L. Nyholm, *J. Power Sources* **2016**, *321*, 185.

[5] J. L. Shi, Y. G. Xia, Z. Z. Yuan, H. S. Hu, X. F. Li, H. Jiang, H. M. Zhang, Z. P. Liu, *J. Mater. Chem. A* **2015**, *3*, 7006.

[6] J. Dai, C. Shi, C. Li, X. Shen, L. Peng, D. Wu, D. Sun, P. Zhang, J. Zhao, *Energy Environ. Sci.* **2016**, *9*, 3252.

[7] A. Banerjee, B. Ziv, Y. Shilina, S. Luski, I. C. Halalay, D. Aurbach, *Adv. Energy Mater.* **2016**, *7*, 1601556.

[8] M. Chi, L. Shi, Z. Wang, J. Zhu, X. Mao, Y. Zhao, M. Zhang, L. Sun, S. Yuan, *Nano Energy* **2016**, *28*, 1.

[9] Y. Zhai, K. Xiao, J. Yu, B. Ding, *J. Power Sources* **2016**, *325*, 292.

[10] W. Chen, L. Shi, Z. Wang, J. Zhu, H. Yang, X. Mao, M. Chi, L. Sun, S. Yuan, *Carbohyd. Poly.* **2016**, *147*, 517.

[11] J. H. Kim, M. Gu, H. Lee do, J. H. Kim, Y. S. Oh, S. H. Min, B. S. Kim, S. Y. Lee, *Nano Lett.* **2016**, *16*, 5533.

[12] C. Liu, Z. Shao, J. Wang, C. Lu, Z. Wang, *RSC Adv.* **2016**, *6*, 97912.

[13] W. Na, A. S. Lee, J. H. Lee, S. S. Hwang, E. Kim, S. M. Hong, C. M. Koo, *ACS Appl. Mater. Interfaces* **2016**, *8*, 12852.

[14] W. Chen, L. Shi, H. Zhou, J. Zhu, Z. Wang, X. Mao, M. Chi, L. Sun, S. Yuan, *ACS Sus. Chem. Eng.* **2016**, *4*, 3794.

[15] J.-H. Kim, J.-H. Kim, J.-M. Kim, Y.-G. Lee, S.-Y. Lee, *Adv. Energy Mater.* **2015**, *5*, 1500954.

[16] F. Jiang, Y. Nie, L. Yin, Y. Feng, Q. Yu, C. Zhong, *J. Mem. Sci.* **2016**, *510*, 1.

[17] Y. S. Oh, G. Y. Jung, J. H. Kim, J. H. Kim, S. H. Kim, S. K. Kwak, S. Y. Lee, *Adv. Funct. Mater.* **2016**, *26*, 7074.

[18] M.-H. Ryou, Y. M. Lee, J.-K. Park, J. W. Choi, *Adv. Mater.* **2011**, *23*, 3066.

[19] D.-W. Kim, J.-M. Ko, J.-H. Chun, S.-H. Kim, J.-K. Park, *Electrochem. Commun.* **2001**, *3*, 535.

[20] D. Lin, D. Zhuo, Y. Liu, Y. Cui, *J. Am. Chem. Soc.* **2016**, *138*, 11044.

[21] M. Raja, N. Angulakshmi, S. Thomas, T. P. Kumar, A. M. Stephan, *J. Mem. Sci.* **2014**, *471*, 103.

[22] F. Jiang, L. Yin, Q. Yu, C. Zhong, J. Zhang, *J. Power Sources* **2015**, *279*, 21.

[23] Z. Wang, F. Guo, C. Chen, L. Shi, S. Yuan, L. Sun, J. Zhu, *ACS Appl. Mater. Interfaces* **2015**, *7*, 3314.

[24] Y.-C. Jung, S.-K. Kim, M.-S. Kim, J.-H. Lee, M.-S. Han, D.-H. Kim, W.-C. Shin, M. Ue, D.-W. Kim, *J. Power Sources* **2015**, *293*, 675.

[25] I. Kuribayashi, *J. Power Sources* **1996**, *63*, 87.

[26] L. C. Zhang, X. Sun, Z. Hu, C. C. Yuan, C. H. Chen, *J. Power Sources* **2012**, *204*, 149.

[27] B. Zhang, Q. F. Wang, J. J. Zhang, G. L. Ding, G. J. Xu, Z. H. Liu, G. L. Cui, *Nano Energy* **2014**, *10*, 277.
